# Reduction in membranous expression of **β**-catenin and increased cytoplasmic E-cadherin expression predict poor survival in gastric cancer

**DOI:** 10.1038/sj.bjc.6693437

**Published:** 1999-12

**Authors:** S Ramesh, J Nash, P G McCulloch

**Affiliations:** Departments of Surgery andPathology, University of Liverpool, 5th Floor, UCD, Daulby Street, Liverpool L69 3GA, UK

**Keywords:** gastric cancer, beta-catenin and survival

## Abstract

β-catenin, a component of the E-cadherin–catenin cell adhesion complex, also plays a separate intracellular signalling role, interacting with APC protein. Intracellular accumulation of β-catenin is common in colorectal neoplasia. β-catenin abnormalities are associated with poor survival in gastric cancer, but previous studies do not differentiate between membrane-associated and intracellular β-catenin. In this study we aimed to determine which type of expression abnormalities for E-cadherin, β-catenin and α-catenin correlate with clinico-pathological features and survival in gastric cancer. Immunoperoxidase staining of paraffin-embedded sections from 40 gastric cancers was performed for E-cadherin, α- and β-catenins using microwave unmasking and an avidin–biotin technique. Clinical data were obtained from case records and cancer registry records. Reduced membranous expression of β-catenin occurred in 10/12 (83%) diffuse and 8/28 (29%) intestinal tumours (*P* = 0.0014), and was associated with poor differentiation (*P* = 0.0015) and short survival (*P* = 0.032), but not with age, sex, tumour size or nodal status. Nuclear expression of β-catenin was uncommon; cytoplasmic expression was observed in 13/40 cases (33%) but did not correlate with histology, tumour grade or survival. Reduced E-cadherin membrane expression was associated with lymph node metastasis (*P* = 0.02). Neither E-cadherin or α-catenin expression correlated with survival. Reduced membranous expression of β-catenin predicts poor prognosis in gastric cancer, whilst ectopic intracellular expression is relatively rare. The apparent differences in β-catenin expression from those found in colon cancer merit further study. © 1999 Cancer Research Campaign


*British Journal of Cancer* (1999) **81**(8), 1392–1397
© 1999 Cancer Research Campaign
Article no. bjoc.1999.0857

### Reduction in membranous expression of β-catenin and increased cytoplasmic E-cadherin expression predict poor survival in gastric cancer

**S Ramesh[1], J Nash[2] and PG McCulloch[1]**

Departments of [1]Surgery and [2]Pathology, University of Liverpool, 5th Floor, UCD, Daulby Street, Liverpool L69 3GA, UK

**Summary** β-catenin, a component of the E-cadherin–catenin cell adhesion complex, also plays a separate intracellular signalling role, interacting with APC protein. Intracellular accumulation of β-catenin is common in colorectal neoplasia. β-catenin abnormalities are associated with poor survival in gastric cancer, but previous studies do not differentiate between membrane-associated and intracellular β-catenin. In this study we aimed to determine which type of expression abnormalities for E-cadherin, β-catenin and α-catenin correlate with clinico-pathological features and survival in gastric cancer. Immunoperoxidase staining of paraffin-embedded sections from 40 gastric cancers was performed for E-cadherin, α- and β-catenins using microwave unmasking and an avidin–biotin technique. Clinical data were obtained from case records and cancer registry records. Reduced membranous expression of β-catenin occurred in 10/12 (83%) diffuse and 8/28 (29%) intestinal tumours ($P = 0.0014$), and was associated with poor differentiation ($P = 0.0015$) and short survival ($P = 0.032$), but not with age, sex, tumour size or nodal status. Nuclear expression of β-catenin was uncommon; cytoplasmic expression was observed in 13/40 cases (33%) but did not correlate with histology, tumour grade or survival. Reduced E-cadherin membrane expression was associated with lymph node metastasis ($P = 0.02$). Neither E-cadherin or α-catenin expression correlated with survival. Reduced membranous expression of β-catenin predicts poor prognosis in gastric cancer, whilst ectopic intracellular expression is relatively rare. The apparent differences in β-catenin expression from those found in colon cancer merit further study. © 1999 Cancer Research Campaign

**Keywords**: gastric cancer; beta-catenin and survival

Despite a steady decline in incidence, gastric carcinoma remains the second most common lethal malignancy worldwide (Whelan et al, 1993), and causes 10 000 deaths per year in England and Wales (Cancer Research Council Factsheet 18). Infection with *Helicobacter pylori* has been strongly implicated in its pathogenesis (Eurogast Study Group, 1993), as have dietary factors (Buiatti et al, 1989), but the molecular mechanisms underlying its development remain relatively poorly understood. Numerous abnormalities of expression have been reported in molecules modulating growth and cell division such as tyrosine kinase growth factor receptors (Tahara et al, 1986), p53 and other apoptosis-related genes (Hollstein et al, 1991) and more recently genes controlling intercellular adhesion, such as E-cadherin and related molecules. Numerous reports now indicate a role for disruption of the cadherin–catenin complex in a variety of human cancers (Bringuier et al, 1993; Oka et al, 1993; Pignatelli et al, 1994; Krishnadath et al, 1997). Experimental studies suggest an important permissive role for loss of cadherin complex function in invasion and metastasis (Takeichi, 1993; Birchmeier et al, 1994). Links have been revealed between the E-cadherin–catenin complex and intracellular signalling pathways involving the tumour suppressor gene APC (Rubinfeld et al, 1993; Munemitsu et al, 1995) and the oncogene wnt-1 (Papkoff et al, 1996; Korinek et al, 1997). More recent work has confirmed that tyrosine phosphorylation of β-catenin may participate in regulation of the cadherin–catenin complex in vivo (Takayama et al, 1998). Abnormal expression of each of the main components of the complex (E-cadherin, α-, β-catenin and plakoglobin) have been demonstrated in gastric cancer, and are commoner in the diffuse, poorly differentiated than in the intestinal, more well-differentiated histological type (Jawahari et al, 1997). Reduced expression of β-catenin has been shown to be an independent predictor of poor prognosis in gastric cancer (Jawahari et al, 1997), but the nature of the expression abnormalities, and their specific associations with survival have not been adequately evaluated. In colorectal cancer, increased cytoplasmic and nuclear staining is an independent predictor of poor survival (Hugh et al, 1999), whereas loss of expression at the cell membrane is not. In the mouse model of familial adenomatous polyposis, cytoplasmic accumulation of β-catenin occurs in the premalignant stage of the adenoma:carcinoma sequence enabling genes associated with neoplastic growth to be overexpressed (Clark et al, 1999). Cytoplasmic and nuclear accumulation of β-catenin are known to result from loss-of-function mutations of the APC gene (Munemitsu et al, 1995), which are common in sporadic colorectal cancer, but less frequent in gastric cancer (Nakatsuru et al, 1992; Powell et al, 1992). It therefore seems that abnormalities of β-catenin expression in gastric cancer may arise by different mechanisms. Detailed study of the expression of β-catenin and other members of the cadherin/catenin complex, and their association with outcome, may therefore yield useful insights into the mechanisms of development and progression of both gastric and colorectal cancer.

*Received 14 January 1999*
*Revised 20 April 1999*
*Accepted 13 May 1999*

*Correspondence to:* PG McCulloch

### MATERIALS AND METHODS

### Tumour specimens

Formalin-fixed, paraffin-embedded gastric carcinoma tissue samples were obtained from 48 consecutive patients undergoing partial or total gastrectomy for gastric carcinoma between 1992 and 1995 from the archival tissue of the Pathology Department at Aintree NHS Trust. Adjacent non-involved gastric mucosa was obtained from all cases. Of the 48 patients identified, 40 were considered suitable for analysis (31 male patients, median age 68 years, range 57–87 years). Five cases were deemed ineligible because follow-up data was unavailable (two patients had moved out of the region, three had died from early post-operative complications) and suitable well preserved blocks could not be obtained in three cases. Tumours were classified using the Lauren system (Lauren, 1965) into intestinal and diffuse types. Intestinal type tumours were graded into well, moderately or poorly differentiated according to the predominant pattern of the tumour. Tumours were staged using the criteria for TNM evaluation of the unified international gastric cancer staging classification (Maruyama and Miwa, 1987).

### Clinical details

Clinico-pathological information and survival data were obtained from hospital records, contact with general practitioners and the Cancer Registry office. The data collected in each case is shown in Table 1.

**Table 1** Clinico-pathological data collected

| |
|---|
| Age |
| Sex |
| T stage |
| N stage |
| M stage |
| UICC stage |
| Histological grade |
| Lauren class |
| Disease free survival |
| Overall survival |

### Antibodies (monoclonal)

Mouse monoclonal immunoglobulin (Ig)G antibody to E-cadherin (HECD 1) was purchased from R&D Systems Europe, Abingdon, UK. Anti-β-catenin monoclonal IgG antibody, and anti-α-catenin monoclonal antibody were bought from Affinity Research Products Ltd (Exeter, UK). Final antibody dilutions, determined by serial dilutions against positive and negative controls, were: anti-E-cadherin 1:100, anti-β-catenin 1:75 and anti-α-catenin 1:20.

### Immunostaining

Five-micrometre sections were cut from formalin-fixed, paraffin-embedded tissue blocks for haematoxylin and eosin (H&E) and immunostaining. Consecutive slides for assessment of Lauren class, grade and immunostaining were taken from the block containing the greatest vertical depth of penetration of the gastric wall by the tumour. Slides for evaluation of grade and Lauren class were stained with H&E in conventional fashion. Sections for immunohistochemical staining were mounted onto poly-L lysine-coated slides.

A standard avidin–biotin immunoperoxidase technique was used. Sections were dewaxed using xylene and transferred to alcohol. Endogenous peroxidase activity was quenched by incubation in 3% hydrogen peroxide in methanol for 20 min. Antigen retrieval was by microwaving for 15 min at 674 W in citrate buffer at pH 6.0. Non-specific binding of secondary antibody was blocked by incubating with 100 μl of fetal calf serum (FCS) diluted to 1:20 for 10 min in a Shandon Sequenza tray (Shandon Southern Ltd, UK). Incubation with primary antibody was at 37°C for 120 min for anti-E-cadherin, 60 min at 37°C for anti-β-catenin, and 4°C overnight for anti-α-catenin. After three washes with Tris-buffered saline (TBS), the slides were incubated at room temperature for 45 min with biotinylated secondary sheep anti-mouse antibody (Amersham Life Science, Little Chalfont, Buckinghamshire, UK) diluted 1:200 in TBS. Following three further washings in TBS the slides were incubated with avidin–biotin complex (ABC)/horseradish peroxidase solution (Dako, UK) diluted to 1:100 with TBS. The slides were washed further in TBS, and developed in activated 3,3-diaminobenzidine-tetrahydrochloride containing 0.01% hydrogen peroxide for 8 min, and the reaction stopped in water. The slides were counterstained with haematoxylin and dehydrated in alcohol prior to mounting. Normal colonic epithelium was used as positive control and adjacent normal gastric mucosa as internal control. Negative controls included adjacent sections of the same block in which the primary antibody was replaced by non-specific mouse IgG.

### Interpretation of staining

Slides were independently examined by two experienced observers (SR and JN) who were blinded to the stage of the tumour and to the initial score of the other observer. The intensity (absent, weak or strong) was scored in a semi-quantitative fashion graded 0–2, and pattern (membranous, cytoplasmic or nuclear) of staining was recorded. In cases of heterogenous staining (more than 10% variation) within the tumour, particularly related to variations in the degree of differentiation, the score was based on the dominant pattern. Where there were differences, a consensus decision on the final score was reached after joint re-examination of the slides and discussion.

### Statistical methods

Associations between antigen expression and clinico-pathological indices were examined using the $\chi^2$ test. Survival curves were computed as described by Kaplan and Meier; the log-rank test was used to examine the difference between curves. A $P$-value of $< 0.05$ was accepted as statistically significant. All statistical analyses were performed using the StatView package (Abacus Concepts, Berkley, CA, USA).

### RESULTS

### Control samples and normal mucosa

All colonic control samples and internal control normal adjacent gastric mucosa showed intense membranous staining throughout the epithelium (Figure 1A). Increased intensity of membranous

© 1999 Cancer Research Campaign 

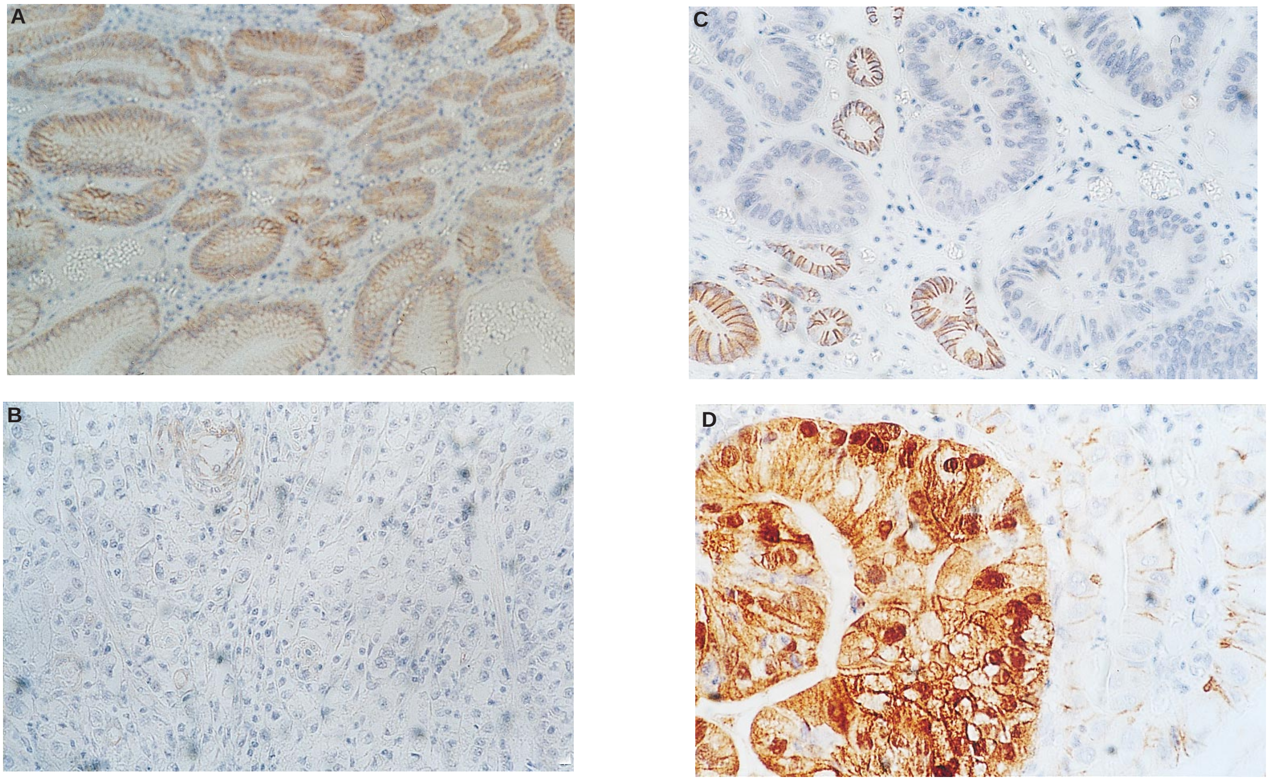

**Figure 1** Photomicrographs to show immunohistochemical staining patterns. (**A**) Normal gastric mucosa to show strong membranous β-catenin expression, with no intracellular expression. (**B**) Absent membranous β-catenin expression in diffuse gastric cancer. (**C**) Absent membranous E-cadherin expression in gastric cancer. (**D**) Strong nuclear β-catenin expression in intestinal type cancer

**Table 2** Relationship between immunostaining[a] and histology

| | E-Cadherin | | | | | | β-catenin | | | | | |
|---|---|---|---|---|---|---|---|---|---|---|---|---|
| | Memb | | Cyto | | Nucl | | Memb | | Cyto | | Nucl | |
| | diff | int | diff | int | diff | int | diff | int | diff | int | diff | int |
| Strong | 3 | 7 | 2 | 9 | 0 | 0 | 2 | 20 | 3 | 10 | 1 | 4 |
| Weak | 9 | 21 | 10 | 19 | 40 | 40 | 10 | 8 | 9 | 18 | 11 | 24 |
| $\chi^2$ *P*-value | NS | | NS | | NS | | 0.0014 | | NS | | NS | |

Memb = membranous, cyto = cytoplasm, nucl = nuclear, diff = diffuse cancer, int = intestinal type cancer. aData for α-catenin not shown.

staining was noted in the deeper parts of antral, body and cardiac glands.

### Carcinoma

Forty gastric cancers were studied by immunohistochemistry [IHC; intestinal type, 28 (70%); diffuse, 12 (30%)] according to Lauren classification of gastric cancer. Tumour differentiation was graded into well- ($n$ = 1, 2.5%), moderate ($n$ = 19, 47.5%) and poor ($n$ = 20, 50%). For purposes of statistical analysis, well- and moderately differentiated tumours were grouped together. All diffuse cancers were classed as poorly differentiated.

### Tumour staining vs histology

### *Membranous*

Reduced or absent membranous expression of β-catenin was more common than ectopic intracellular expression. Weak or absent membranous β-catenin expression was found in 18 of 40 cases (45%): ten of 12 (83.4%) diffuse and in eight of 28 (28.6%) intestinal type cancers ($P$ = 0.0014, see Figure 1B). In this series, no difference between E-cadherin membranous staining patterns in the two tumour types was found, with loss of membranous expression of E-cadherin in nine of 12 (75%) diffuse cancers and in 21 of 28 (75%) intestinal type cancers (Figure 1c and Table 2). α-catenin staining patterns equally were unrelated to tumour type, with loss of membranous expression in 11 of 12 (91%) diffuse cancers and 22 of 28 (78.6%) of intestinal type cancers (results not shown).

### *Intracellular*

Three of 12 diffuse tumours and ten of 28 intestinal tumours showed strong cytoplasmic β-catenin staining. Four intestinal tumours (Figure 1D) and one diffuse tumour showed strong β-catenin nuclear staining. These differences were not statistically significant. For neither E-cadherin nor α-catenin was there any

*© 1999 Cancer Research Campaign*

**Table 3** Relationship between immunostaining[a] and tumour differentiation

| | **E-Cadherin** | | | | | | **β-catenin** | | | | | |
|---|---|---|---|---|---|---|---|---|---|---|---|---|
| | **Memb** | | **Cyto** | | **Nucl** | | **Memb** | | **Cyto** | | **Nucl** | |
| | **mod** | **poor** | **mod** | **poor** | **mod** | **poor** | **mod** | **poor** | **mod** | **poor** | **mod** | **poor** |
| Strong | 7 | 3 | 9 | 2 | 0 | 0 | 16 | 6 | 8 | 5 | 3 | 2 |
| Weak | 13 | 17 | 11 | 18 | 40 | 40 | 4 | 14 | 12 | 15 | 17 | 18 |
| $\chi^2$ *P*-value | NS | | 0.0132 | | NS | | 0.0015 | | NS | | NS | |

Memb = membranous, cyto = cytoplasm, nucl = nuclear, mod = moderately differentiated gastric carcinoma. [a]Data for α-catenin not shown.

**Table 4** Relationship between immunostaining[a] and nodal status

| | **E-Cadherin** | | | | | | **β-catenin** | | | | | |
|---|---|---|---|---|---|---|---|---|---|---|---|---|
| | **Memb** | | | **cyto** | | | **Memb** | | | **cyto** | | |
| | **N0** | **N1** | **N2/3** | **N0** | **N1** | **N2/3** | **N0** | **N1** | **N2/3** | **N0** | **N1** | **N2/3** |
| Strong | 5 | 0 | 5 | 5 | 1 | 5 | 6 | 7 | 10 | 4 | 2 | 7 |
| Neg | 4 | 10 | 16 | 4 | 9 | 16 | 3 | 3 | 13 | 5 | 8 | 14 |
| χ2 P-value | | 0.0199 | | | 0.07 | | | NS | | | NS | |

Memb = membranous, cyto = cytoplasm, nucl = nuclear, neg = negative. aData for α-catenin not shown.

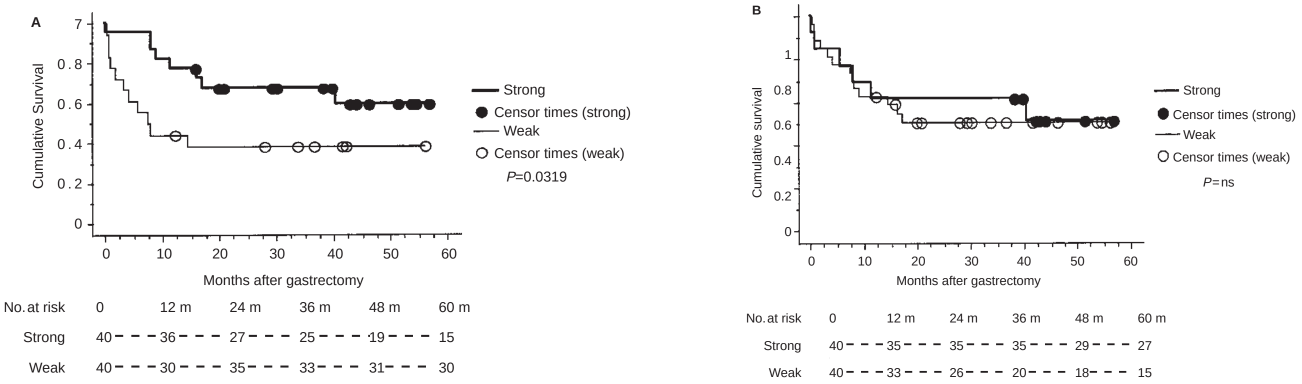

**Figure 2** (**A**) Kaplan–Meier survival curves showing a statistically significant survival advantage in tumours with strong membranous β-catenin expression (thick line), compared with those that showed weak membranous expression (thin line). (**B**) Kaplan–Meier survival curves showing no differences in survival in tumours showing strong cytoplasmic β-catenin expression (thick line), compared with those that showed weak cytoplasmic expression (thin line)

correlation between intracellular staining and tumour histology (Table 2) (results for α-catenin not shown).

### Tumour staining vs differentiation

### *Membranous*

Abnormalities of membranous expression of β-catenin appeared to be related to tumour grade. Loss of membranous expression was seen in four of 20 moderately differentiated cancers but in 14 of 20 poorly differentiated cancers ($P < 0.0015$) (Table 3). For E-cadherin and α-catenin there was no correlation between membranous staining and degree of tumour differentiation (Table 3) (results for α-catenin not shown).

### *Intracellular staining*

Abnormalities of intracellular cytoplasmic expression of E-cadherin correlated with tumour grade. Strong cytoplasmic E-cadherin expression was found in nine of 20 moderately differentiated tumours, compared to two of 20 poorly differentiated tumours ($P = 0.0132$) (Table 3). Nuclear localization of β-catenin was observed in three of 20 moderately differentiated, compared to two of 20 poorly differentiated tumours. Nuclear expression was not observed for E-cadherin or α-catenin immunostaining.

### Tumour staining vs nodal status

Lymph node status was categorized according to TNM rules (Sobin and Wittekind, 1997). For purposes of statistical analysis N2 and N3 were grouped together.

The relationship between N stage and expression of E-cadherin and β-catenin molecules at the membrane and in cytoplasm is shown in Table 4. Only the association between E-cadherin membrane staining and nodal status was significant. Of 21 N2/3 stage tumours, 16 (76%) showed a loss of membranous E-cadherin staining ($P = 0.0199$). Nuclear expression of β-catenin was seen in

*© 1999 Cancer Research Campaign*

one patient with N0 nodal status, and two each of N1 and N2/3 status. For brevity, the nuclear expression is not shown in Table 4.

### Survival analysis

Kaplan–Meier curves were computed to compare survival of patients with normal versus reduced membranous expression, and normal versus abnormal intracellular expression (both cytoplasmic and nuclear) for each of the three molecules. Neither membranous nor intracellular abnormalities of E-cadherin or α-catenin showed any association with survival. The overall median survival of patients in this study was 18.3 months (range 0.13–56.6). Median survival for patients showing *any* abnormality of β-catenin expression (membranous or cytoplasmic) was 12.0 months. Median survival was 30.0 months in the normal and 7.4 months in the reduced membranous expression group. Loss of membranous β-catenin expression was associated with significantly decreased survival ($P$ = 0.0319, Kaplan–Meier curves, Figure 2A). The survival of patients with abnormal cytoplasmic (Figure 2B) or nuclear expression of β-catenin was not significantly different from that of patients without this abnormality.

### DISCUSSION

Mutations affecting intercellular adhesion mechanisms have emerged as important steps in the development and progression of many human epithelial tumours. Loss of E-cadherin expression correlates with advanced stage and high grade in cancers of the breast (Oka et al, 1993), stomach (Shimoyama et al, 1991; Matsuura et al, 1992), colorectum (Nigam et al, 1993), pancreas (Pignatelli et al, 1994), bladder (Bringuier et al, 1993) and prostate (Umbas et al, 1992).

In stomach cancer, mutations of the E-cadherin gene have been reported in diffuse and histologically indeterminate tumours, but seem rare in intestinal type cancer (Becker and Hofler, 1995). A germline mutation in E-cadherin associated with familial gastric cancer was recently reported in a New Zealand kindred (Guilford et al, 1998). Mutations in α- and β-catenins have not been convincingly demonstrated, but protein expression abnormalities are relatively frequent, and occur in both diffuse and intestinal cancers (Oka et al, 1992; Matsui et al, 1994; Jawhari et al, 1997). We have confirmed a high frequency of expression abnormalities for all three molecules studied. Analysis of the type of expression revealed somewhat different patterns from previous reports, which considered only global abnormality versus normality. Surprisingly, E-cadherin expression overall did not correlate with grade, although ectopic cytoplasmic expression of E-cadherin did. β-catenin expression was closely related to tumour grade and differentiation, loss of membranous expression occurring much more frequently in poorly differentiated tumours. We found that β-catenin but not α-catenin or E-cadherin expression abnormalities were associated with poor survival, confirming the findings of Jawhari et al (1997), but contradicting earlier reports (Yonemura et al, 1995; Gabbert et al, 1996). Analysis of the type of expression revealed that loss of membranous expression, rather than ectopic cytoplasmic expression, was correlated with poor survival, the opposite of the situation recently reported in colon cancer (Hugh et al, 1999).

Abnormal protein expression of the components of the cadherin–catenin complex may occur for a number of reasons. Ectopic or reduced expression may occur directly because of mutations in the gene concerned (Becker and Hofler, 1995), or indirectly due to alterations in one of its partners in the complex leading, for example, either to interference with the intermolecular binding which anchors the protein in the complex (Kawanishi et al, 1995; Streit et al, 1996) or altered transcription of their genes. Abnormal expression of β-catenin can occur due to tyrosine phosphorylation induced by activated growth factor receptors (Shibamoto et al, 1994).

Given this complex picture, it is difficult to draw firm conclusions about the specific molecular events which are occurring from immunohistochemical studies alone. We found that loss of membranous β-catenin expression, but not gain of intracellular expression, was significantly associated with poor survival. Therefore dysfunction of the adhesion complex may influence progression more than changes in signalling pathways brought about by accumulation of intracellular β-catenin.

The importance of cadherins in mediating intercellular adhesion suggests that loss of their function might promote invasion and metastasis. Evidence for such a role has been provided by experiments in vitro and in animal models (see above). Recently, β-catenin has been shown to play a distinct and separate role in intracellular signalling in which it complexes with the APC protein (Rubinfeld et al, 1995), and thereby becomes degraded (Munemitsu et al, 1995). Adenomas and carcinomas arising in patients with familial polyposis carry APC mutations which prevent binding and degradation of β-catenin, and in these tumours, intracellular accumulation of the protein occurs (Inomata et al, 1996). Free β-catenin binds the transcription factors Tcf and Lef, assisting the up-regulation of transcription (Behrens et al, 1996; Korinek et al, 1997; Morin et al, 1997). Intense current interest continues in the possibility that the tumour suppressor role of APC may be mediated through its interaction with β-catenin, which may thereby have an important role in signalling mechanisms such as transduction of signals from the wnt-1 oncogene (Hinck et al, 1994).

The differences between our findings in gastric cancer and those reported by Hugh et al in colorectal cancer need to be confirmed in a direct comparison, since they suggest that different mechanisms may be important in progression in the two tumour types. This is particularly interesting in view of the well known differences in natural history between gastric and colonic cancer. Recurrence after apparently curative resection is not uncommon in both tumours, but the patterns of failure are strikingly different. In colorectal cancer, isolated liver metastasis without evidence of metastatic disease elsewhere occurs in about 25% of complete resections (Welch and Donaldson, 1979), but this very rarely happens in gastric cancer (Ochiai et al, 1994). We speculate that ectopic expression of β-catenin may be associated with enhanced blood-borne metastasis, whereas loss of membranous expression is associated with enhanced local growth and spread. Further studies are required to test the hypothesis that blood-borne metastasis is associated with a particular type of β-catenin expression abnormality.

### REFERENCES

Becker KF and Hofler H (1995) Frequent somatic allelic inactivation of the E-cadherin gene in gastric carcinomas. *J Natl Cancer Inst* **87**: 1082–1084

Behrens J, Kries JP, Kuhl M, Bruhn L, Wedlich D, Grosschedl R and Birchmeier W (1996) Functional interaction of β-catenin with the transcription factor LEF-1. *Nature* **382**: 638–642

© 1999 Cancer Research Campaign

Birchmeier W and Behrens J (1994) Cadherin expression in carcinomas – role in the formation of cell-junctions and the prevention of invasiveness. *Biochim Biophys Acta Rev Cancer* **1198**: 11–26

Bringuier PP, Umbas R, Schaafsma HE, Karthus HF, Debruyne FM and Schalken JA (1993) Decreased E-cadherin immunoreactivity correlates with poor survival in patients with bladder tumours. *Cancer Res* **53**: 3241–3248

Buiatti E, Palli D, DeCarli A et al (1989) A case–control study of gastric cancer and diet in Italy. *Int J Cancer* **44**: 611–616

Cancer Research Campaign (1993) *Factsheet 18*. Cancer Research Campaign: London

Clark DJ, Scholefield JH and Watson SA (1999) Comparison of E-Cadherin and β-catenin expression in a mouse model of famalial adenomatous polyposis. *Gut* **44**: A98

Eurogast Study Group (1994) An international association between *Helicobacter pylori* infection and gastric cancer. *Lancet* **34**: 1359–1362

Gabbert HE, Meullers W, Schneiders A, Meier S, Moll R, Birchmeier W and Hommel G. Prognostic value of E-cadherin expression in 413 gastric carcinomas. *Int J Cancer* **69**: 184–189

Guilford P, Hopkins J, Harraway J, MacLeod M, MacLeod N et al (1998) E-cadherin germline mutations in familial gastric cancer. *Nature* **392**: 402–405

Hinck L, Nelson WJ and Papkoff J (1994) WNT-1 modulates cell–cell adhesion in mammalian cells by stabilizing beta-catenin binding to the cell adhesion protein cadherin. *J Cell Biol* **124**: 729–741

Holstein M, Sidransky D, Vogelstein B and Harris CC (1991) p53 mutations in human cancers. *Science* **253**: 49–53

Hugh TJ, Dillon SA, Poston GJ, Taylor BA, Pignatelli M and Kinsella AN (1999) Cadherin-catenin expression in primary colorectal cancer: a survival analysis. *Br J Cancer* **80**: 1046–1051

Inomata M, Ochiai A, Akimoto S, Kitano S and Hirohashi S (1996) Alteration of beta-catenin expression in colonic epithelial cells of familial adenomatous polyposis patients. *Cancer Res* **56**: 2213–2217

Jawhari A, Jordan S, Poole S, Browne P, Pignatelli M and Farthing MJG (1997) Abnormal immunoreactivity of the E–cadherin–catenin complex in gastric cancer: relationship with patient survival. *Gastroenterology* **112**: 46–54

Kawanishi J, Kato J, Sasaki K, Fujii S, Watanabe N and Niitsu Y (1995) Loss of E-cadherin-dependent cell–cell adhesion due to mutation of the beta-catenin gene in a human cancer cell line, HSC-39. *Mol Cell Biol* **15**: 1175–1181

Korinek V, Barker N, Morin PJ, Van Wichen D, de Weger R, Kinzler KW, Vogelstein B and Clevers H (1997) Constitutive transcriptional activation by a β-catenin-Tcf complex in APC−/−colon carcinoma. *Science* **275**: 1784–1787

Krishnadath KK, Tilanus HW, van Blankenstein M, Hop WCJ, Kremers ED, Dinjens WNM and Bosman FT (1997) Reduced expression of the cadherin–catenin complex in oesophageal adenocarcinoma correlates with poor prognosis. *J Pathol* **182**: 331–338

Lauren P (1965) The two histological main types of gastric carcinoma. *Acta Pathol Microbiol Scand* **64**: 31–49

Maruyama K and Miwa K (1987) Japanese staging system for gastric cancer: evaluation and documentation of tumour extension. *Scand J Gastroenterol* **22**: 22–26

Matsui S, Shiozaki H, Inoue M, Tamura S, Doki Y, Kadowaki T, Iwazana T, Shimaya K, Nagafuchi A, Tsukita S and Mori T (1994) Immunohistochemical evaluation of alpha-catenin in human gastric cancer. *Virchows Archiv Int J Pathol* **424**: 375–381

Matsuura K, Kawanishi J, Fujii S, Imamura M, Hirano S, Takeichi M and Niitsu Y (1992) Altered expression of E-cadherin in gastric cancer tissue and carcinomatous fluid. *Br J Cancer* **66**: 1122–1130

Morin PJ, Sparks AB, Korinek V, Barker N, Clevers H, Vogelstein B and Kinzler KW (1997) Activation of β-catenin-Tcf signalling in colon cancer by mutations in β-catenin or APC. *Science* **275**: 1787–1790

Munemitsu S, Albert I, Souza B, Rubinfeld B and Polakis P (1995) Regulation of intracellular β-catenin levels by the adenomatous polyposis-coli (APC) tumor-suppressor protein. *Proc Natl Acad Sci USA* **92**: 3046–3050

Nakatsuru S, Yanagisawa A, Ichii S, Tahara E, Kato Y, Nakamura Y and Horii A (1992) Somatic mutations of the *APC* gene in gastric cancer: frequent mutations in very well-differentiated adenocarcinoma and signet-ring cell carcinoma. *Hum Mol Gen* **1**: 559–563

Nigam AK, Savage FJ, Boulos PB, Stamp GW, Liu D and Pignatelli M (1993) Loss of cell–cell and cell matrix adhesion molecules in colorectal cancer. *Br J Cancer* **68**: 507–514

Ochiai T, Sasako M, Mizuno S, Kinoshita T, Takayama T et al (1994) Hepatic resection for metastatic tumours from gastric cancer: analysis of prognostic factors. *Br J Surg* **81**: 1175–1178

Oka H, Shiozaki H, Kobayashi K, Tahara H, Tamura S et al (1992) Immunohistochemical evaluation of E-cadherin adhesion molecule expression in human gastric cancer. *Virchows Arch [A]* **421**: 149–156

Oka H, Shiozaki H, Kobayashi K, Inoue M, Tahara E, Kobayashi T, Takatsuka Y, Matsuyoshi N, Hirano S and Takeichi M (1993) Expression of E-cadherin cell adhesion molecules in human breast cancer tissues and its relationship to metastasis. *Cancer Res* **53**: 1696–1701

Papkoff J, Rubinfeld B, Schryver B and Polakis P (1996) Wnt-1 regulates free pools of catenins and stabilizes APC–catenin complexes. *Mol Cell Biol* **16**: 2128–2134

Pignatelli M, Ansari TW, Gunter P, Liu D, Hirano S, Takeichi M, Kloppel G and Lemoine NR (1994) Loss of membranous E-cadherin expression in pancreatic cancer: correlation with lymph node metastasis, high grade and advanced stage. *J Pathol* **174**: 243–248

Powell SM, Zilz N, Beazer-Barclay Y, Bryan TM, Hamilton SR, Thibodeau SN, Vogelstein B and Kinzler KW (1992) APC mutations occur early during colorectal tumorigenesis. *Nature (Lond)* **359**: 235–237

Rubinfeld B, Souza B, Albert I, Muller O, Chamberlain SH, Masiarz FR, Munimetsu S and Polakis P (1993) Association of the APC gene product with beta-catenin. *Science* **262**: 1731–1734

Rubinfeld B, Souza B, Albert I, Munimetsu S and Polakis P (1995) The APC protein and cadherin form similar but independent complexes with alpha-catenin, beta-catenin and plakoglobulin. *J Biol Chem* **270**: 5549–5555

Shibamoto S, Hayakawa M, Takeuchi K, Hori T, Oku N, Miyazawa K et al (1994) Tyrosine phosphorylation of beta-catenin and plakoglobin enhanced by hepatocyte growth factor and epidermal growth factor in human carcinoma cells. *Cell Adhesion Commun* **1**: 295–305

Shimoyama Y and Hirohashi S (1991) Expression of E- and P-cadherin in gastric carcinoma. *Cancer Res* **51**: 2185–2192

Sobin LH and Wittekind C (eds) (1997) *International Union against Cancer (UICC). TNM Classification of Malignant Tumours*, 5th edn. Springer: Berlin

Streit M, Schmidt R, Hilgenfeld RU, Thiel E and Kreuser (1996) Adhesion receptors in malignant transformation and dissemination of gastrointestinal tumors. *J Mol Med* **74**: 253–268

Tahara E, Sumiyoshi H, Hata J, Yasui W, Taniyama K, Hayashi T, Nagaye S and Sakamoto S (1986) Human epidermal growth factor in gastric carcinoma as a biologic marker of high malignancy. *Jpn J Cancer Res (Gann)* **77**: 145–152

Takayama T, Shiozaki H, Doki Y, Oka H, Inoue M, Yamamoto M, Tamurs S, Shibamoto S, Ito F and Monden M (1998) Aberrant expression and phosphorylation of β-catenin in human colorectal cancer. *Br J Cancer* **77**: 605–613

Takeichi M (1993) Cadherins in cancer: implications for invasion and metastasis. *Curr Opin Cell Biol* **5**: 806–811

Umbas R, Schalken JA, Aalders TW, Carter BSm, Karthaus HF, Schafsma HE, Debruyue FM and Isaacs WB (1992) Expression of cellular adhesion molecule E-cadherin is reduced or absent in high grade prostate cancer. *Cancer Res* **52**: 5104–5109

Welch JP and Donaldson GA (1979) The clinical correlation of an autopsy study of recurrent colorectal cancer. *Ann Surg* **189**: 496–502

Whelan SL, Parkin DM and Masuyer E (eds) (1993) *Trends in Cancer Incidence and Mortality*. IARC Scientific Publications No. 102. IARC: Lyon

Yonemura Y, Ninomiya I, Kaji M, Sugiyama K, Fujimura T et al (1995) Decreased E-cadherin expression correlates with poor survival in patients with gastric cancer. *Ann Cell Pathol* **8**: 177–190

© 1999 Cancer Research Campaign
